# Probabilistic Models, Compressible Interactions, and Neural Coding

**DOI:** 10.1103/pj4s-g4jc

**Published:** 2026-04-23

**Authors:** Luisa Ramirez, William Bialek, Stephanie E. Palmer, David J. Schwab

**Affiliations:** 1 Institute of Developmental Biology and Neurobiology, Johannes-Gutenberg University Mainz, Mainz, Germany; 2 Departamento de Física, Universidade Federal de Minas Gerais, 31270-901 Belo Horizonte, Minas Gerais, Brasil; 3 Joseph Henry Laboratories of Physics and Lewis–Sigler Institute for Integrative Genomics, Princeton University, Princeton, New Jersey 08544, USA; 4 Initiative for the Theoretical Sciences, The CUNY Graduate Center, City University of New York, 365 Fifth Avenue, New York, New York 10016, USA; 5 Center for Studies in Physics and Biology, Rockefeller University, 1230 York Avenue, New York, New York 10021, USA; 6 Department of Organismal Biology and Anatomy and Department of Physics, The University of Chicago, Chicago, Illinois 60637, USA

## Abstract

In physics we often use very simple models to describe systems with many degrees of freedom, but it is not clear why or how this success can be transferred to the more complex biological context. We consider models for the joint distribution of many variables, as with the combinations of spiking and silence in large networks of neurons. In this probabilistic framework, we argue that simple models are possible if the mutual information between two halves of the system is consistently subextensive, and if this shared information is compressible. These conditions are not met generically, but they are met by real-world data, such as natural images and the activity in a population of retinal output neurons. We introduce compression strategies that combine the information bottleneck with an iteration scheme inspired by the renormalization group, and we find that the number of parameters needed to describe the distribution of joint activity scales with the square of the number of neurons, even though the interactions are not well approximated as pairwise. Our results also show that this shared information is essentially equal to the information that individual neurons carry about natural visual inputs, which has surprising implications for the neural code.

## I. INTRODUCTION

In statistical mechanics, we routinely analyze the joint probability distribution of very large numbers of variables; in field theory this number is infinite, at least formally [1,2]. There is considerable interest in giving a similar probabilistic description outside the traditional domains of physics, spurred in part by the availability of “big data” from a wider variety of complex systems. But the models we consider in most physics problems are highly constrained, and without these constraints we must learn the underlying distribution from the data. If what we observe are discrete states or events, then the probability distribution is a list of numbers, one for each possible outcome, and this number is beyond astronomical: in an image with just N=100 pixels, where each pixel can be black or white, the number of possible images ($2^{N}\sim 10^{30}$) is larger than the age of the universe in seconds. Under these conditions it is physically impossible to “measure” the underlying probability distribution from data alone, and it will continue to be impossible no matter how our technology evolves.

The problem of inferring large probabilistic models has been made more urgent by enormous growth in our ability to monitor, simultaneously, the functional activity of many degrees of freedom in living systems. Examples range from the expression levels of many genes in a single cell [3–6] to the electrical activity of many neurons in the brain [7–13] and the movements of all the individual organisms in a flock or swarm [14]; we emphasize that these are illustrative rather than exhaustive. To understand these experiments we need a theoretical framework that tames the combinatorial explosion of potential complexity.

We can identify several different reactions to the increased dimensionality of the available experimental data. One view, inspired by the success of modern AI, embraces complex models such as deep neural networks, emphasizing that successful predictions are possible even when the number of parameters in our models far exceeds the number of data points [15–17]. The opposite view is that high-dimensional data may lie on lower-dimensional manifolds, so that the search for these manifolds becomes the central problem of data analysis [18,19]. An intermediate approach focuses on the fact that complex models often have a characteristic geometry in parameter space, where some combinations of parameters are essential for successful prediction and others are not [20,21]. Other approaches are more explicitly connected to ideas from statistical physics, including the construction of maximum entropy models that are consistent with low-order correlations [22–28] and the search for scaling behaviors that might point toward models described by a fixed point of the renormalization group [29–32]. The maximum entropy approach has been extended to match global features of the data [26], subsets of higher–order correlations [33], nonlinear transformations of the effective energy [34], or the expectation values of thresholded projections of the data as might be computed by real neurons [35]. For a recent review of statistical physics approaches to networks of real neurons, see [36].

In practice, the search for simplification usually is done by hypothesizing a particular family of models and exploring how far these can take us in the description of real data. We would like to go beyond the exploration of particular models to have more general criteria for the learnability of distributions, in the spirit of theories for the learnability of functions or rules [37,38]. To be concrete, suppose that what we observe is a collection of N binary variables, $\sigma \equiv \left\{\sigma_{1},\sigma_{2},\ldots ,\sigma_{N}\right\}$, so that there are $2^{N}$ possible states, and in general learning the distribution would require many more than $2^{N}$ observations. Can we state conditions on the distribution that are sufficient to guarantee effective learning from a much smaller number of examples, perhaps linear or polynomial in N?^1^ Importantly, are these conditions satisfied in natural data?

Here we suggest that two conditions are sufficient for learnability: the consistent subextensivity of the mutual information between parts of a system, and the compressibility of interactions into an efficient representation. We give general arguments, and test our ideas against statistical physics models, the statistical structure of natural images, and the patterns of electrical activity in the retina as it responds to naturalistic inputs. The implications for the analysis of neural data seem especially rich, and so we explore these in more detail. This paper combines and extends work presented in preliminary form [40,41].

Before proceeding, we admit that our focus on simplified models might seem anachronistic in the era of deep networks. These models, which drive the current revolution in artificial intelligence, are far from simple, in some cases being described by trillions of parameters [42]. In truth, we do not understand the success of these models [43,44]. More relevant for our discussion, these models are still small compared with the number of possible “states” taken on by the relevant variables. For language models, for example, with a five thousand word vocabulary, there are ~10^37^ possible 10-word sequences. While most of these are forbidden by grammatical rules, human level performance requires capturing dependencies across ~100 words [45]. In this context, trillion-parameter models *are* simplified models.

## II. SUBEXTENSITIVITY

If we knew that the N binary variables could be broken down into two independent halves, then the full probability distribution could be written in terms of $2\times 2^{N/2}$ parameters, vastly less than $2^{N}$. More generally, imagine that we can place a bound on the mutual information between the two halves, $I_{1/2}(N)$. If this information is unconstrained, then we need $\sim 2^{N}$ parameters to describe the system, while if $I_{1/2}(N)\to 0$ then we can use only $2\times 2^{N/2}$ and still give an exact description. It seems plausible that if $I_{1/2}(N)$ is sufficiently small, there should be a good approximation that has roughly $2\times 2^{N/2}$ parameters.

Let us call the two halves of our system right and left,

(1) $$\sigma_{R}\equiv \left\{\sigma_{1},\sigma_{2},\ldots ,\sigma_{N/2}\right\},$$

(2) $$\sigma_{L}\equiv \left\{\sigma_{N/2+1},\sigma_{N/2+2},\ldots ,\sigma_{N}\right\}.$$

We recall that the shortest possible code which represents the states σ is based on exact knowledge of the probability distribution, where each state σ is represented by a code word of length L(σ)~-lnP(σ), so that the mean code length is the entropy of the distribution [46,47]. Codes built from approximate models of the distribution will be longer, on average, by an amount ⟨ΔL⟩ equal to the Kullback-Leibler divergence between the model and the true distribution,

(3) $$\langle \Delta L\rangle =\sum_{\sigma }P(\sigma )\left(\left[-{\text{log}}_{2}\;P_{\text{approx}}(\sigma )\right]-\left[-{\text{log}}_{2}\;P(\sigma )\right]\right)\;=\sum_{\sigma }P\left(\sigma \right)\;{\text{log}}_{2}\;\left[\frac{P\left(\sigma \right)}{P_{\text{approx}}\left(\sigma \right)}\right],$$

and this provides a measure of model quality. If our approximate model is the one in which the two halves of the system are independent,

(4) $$P_{\text{approx}}\left(\sigma \right)=P_{R}\left(\sigma_{R}\right)P_{L}\left(\sigma_{L}\right),$$

then this coding cost becomes

(5) $$\langle \Delta L\rangle =\sum_{\sigma }P\left(\sigma_{R},\sigma_{L}\right)\;{\text{log}}_{2}\left[\frac{P\left(\sigma_{R},\sigma_{L}\right)}{P_{R}\left(\sigma_{R}\right)P_{L}\left(\sigma_{L}\right)}\right]$$

(6) $$=I_{1/2}(N)$$

the mutual information between the two halves.

If the variables $\left\{\sigma_{\text{i}}\right\}$ are arranged in real space such that there is a finite correlation length ξ, then the division into right and left halves can be taken literally, and the mutual information between the halves arises from correlations among spins within ξ of the boundary. As a result the mutual information must be related to the area of the boundary, not the volume of the system, and hence is subextensive: if the system is of linear dimension l in d dimensions, we have $N\sim l^{d}$ and $I_{1/2}\sim l^{d-1}$, hence $I_{1/2}(N)=cN^{\alpha }$ with α=1-1/d. In the quantum case this becomes the “area laws” for entanglement [48].

We can ask more generally about systems which, when divided in half, exhibit a mutual information between the halves that behaves as $I_{1/2}(N)=cN^{\alpha }$ with α<1. Then the approximation of the system as two independent halves has a cost that per degree of freedom

(7) $$\frac{\left\langle \Delta L\right\rangle }{N}=cN^{\alpha -1},$$

which vanishes as N becomes large. Thus, subextensive behavior of the mutual information is sufficient to ensure that, for large systems, the reduction in number of parameters from $2^{N}$ down to $2\times 2^{N/2}$ will result in a model that makes only small errors per degree of freedom.

We can now think about cases where the mutual information is *consistently subextensive*, that is when we look at properly chosen pieces of the system with n variables, and cut these pieces in half, we always find a mutual information between the halves $I_{1/2}(n)⩽cn^{\alpha }$. This means that we can keep cutting the variables in half, approximating the distribution as being composed of independent halves, and in the process we make errors that are small when measured as the cost of coding per degree of freedom.

If we make b cuts, then we have

(8) $$\left\langle \Delta L\right\rangle =cN^{\alpha }+2c{\left(\frac{N}{2}\right)}^{\alpha }+4c{\left(\frac{N}{4}\right)}^{\alpha }\;+\ldots +2^{b-1}c{\left(\frac{N}{2^{b-1}}\right)}^{\alpha }$$

(9) $$=cN^{\alpha }\frac{2^{b(1-\alpha )}-1}{2^{1-\alpha }-1}⩽\tilde{c}N^{\alpha }{\left(\frac{N}{n_{0}}\right)}^{1-\alpha },$$

where $\tilde{c}=c/\left(2^{1-\alpha }-1\right)$ and $n_{0}=2^{-b}N$, so that

(10) $$\frac{\left\langle \Delta L\right\rangle }{N}⩽\frac{\tilde{c}}{n_{0}^{1-\alpha }}.$$

This means that we can guarantee a cost ⟨ΔL⟩/N⩽ℓ if we stop cutting once the pieces are of size

(11) $$n_{0}=(\tilde{c}/\ell {)}^{1/(1-\alpha )}.$$

The distribution of $n_{0}$ binary variables requires at most $2^{n_{0}}$ parameters, and this is independent of N. We need one such model for each of the $N/n_{0}$ pieces.

Thus, when the mutual information is consistently subextensive we can make an approximate model that has $P\sim \left(N/n_{0}\right)2^{n_{0}}$ parameters, and the error that we make corresponds to an excess coding cost of ℓ bits per degree of freedom, with $n_{0}$ and ℓ connected through Eq. (11). This number of parameters is linear in the number of degrees of freedom, and hence we expect that the model can be learned from a number of examples which is also linear in the system size.

Thus, we say that a distribution is *learnable* if there is an approximate model whose cost per variable, ⟨ΔL⟩/N, can be made small (⩽ℓ) with a number of parameters that scales at most polynomially in N. The resulting number of parameters is far fewer than the number of possible states. For the connection between learnability and subextensivity to be useful, we require two things: First, it must be that typical probability distributions do *not* have consistently subextensive mutual information. Second, data in interesting systems should exhibit this property.

For any partition of a system into two halves R and L, the mutual information between the two halves is

(12) $$I_{1/2}\equiv I(R;L)=S\left[P_{R}\left(\sigma_{R}\right)\right]+S\left[P_{L}\left(\sigma_{L}\right)\right]-S\left[P\left(\sigma_{R},\sigma_{L}\right)\right].$$

If the variables are binary, and each half contains N/2 degrees of freedom, then the entropy of each half is bounded, $S\left[P_{R}\left(\sigma_{R}\right)\right]⩽N/2$ bits, and similarly for $S\left[P_{L}\left(\sigma_{L}\right)\right]$. Consequently, we can write

(13) $$I_{1/2}⩽N-S\left[P\left(\sigma_{R},\sigma_{L}\right)\right]=N-S(N).$$

**FIG. 1.**
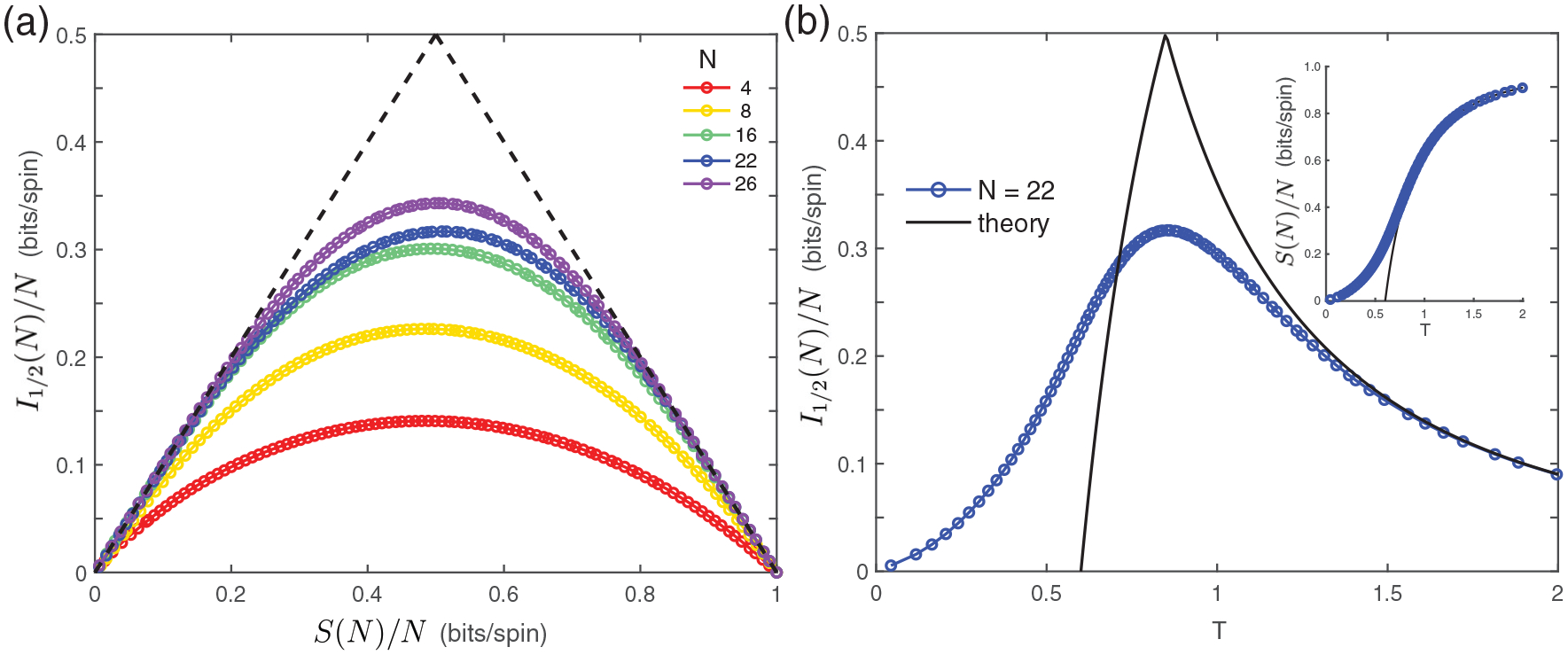
Mutual information between halves of the system for the random energy model. (a) Along each curve at fixed N, we vary T, and compare with the bounds (dashed lines). (b) For N=22, the mutual information between halves of the system versus T. The infinite-size system (solid line) has a cusp in the mutual information, while the entropy (inset) is monotonically increasing with T.

This global upper bound is depicted by the right side of the triangle in Fig. 1.

The mutual information $I_{1/2}$ also cannot be larger than the entropy of either half system, and neither of these entropies can be larger than S(N) itself. This gives the upper bound on the left side of the triangle in Fig. 1. Together with $I_{1/2}⩽N-S(N)$, this gives an allowed triangle in the plot of $I_{1/2}$ versus S(N), as shown in Fig. 1. There are analogous bounds in quantum systems [49].

We define a typical probability distribution to be one in which the probabilities $P\left(\sigma_{R},\sigma_{L}\right)$ are nearly independent random numbers, constrained only by normalization. But then the probability of each state in one half of the system,

(14) $$P_{R}\left(\sigma_{R}\right)=\sum_{\sigma_{L}}P\left(\sigma_{R},\sigma_{L}\right),$$

is the sum of a large number of nearly independent random variables, and from the central limit theorem this should approach its expectation value. This implies that

(15) $$S\left[P_{R}^{\text{typical}}\left(\sigma_{R}\right)\right]\approx S\left[P_{L}^{\text{typical}}\left(\sigma_{L}\right)\right]\approx \frac{N}{2}.$$

Substituting into Eq. (12), we see the *typical* distribution saturates the bound, $I_{1/2}\approx N-S(N)$.

To illustrate this argument, we consider the random energy model (REM), in which each of $2^{N}$ states has an energy drawn at random from a Gaussian distribution with variance $\left\langle E^{2}\right\rangle =N$, with probabilities given by the Boltzmann distribution at temperature T [50]. The states can be labeled by binary numbers and the digits assigned arbitrarily as left and right halves of a spin system. In Fig. 1(a) we show $I_{1/2}(N)$ versus S(N) for these models, with varying T and N, and compare with the bounds derived above. We see that as N increases the information per spin *increases* to approach the bounds, indicating that $I_{1/2}(N)$ is extensive everywhere above the freezing transition. In contrast, models with subextensive mutual information would approach the x-axis in this plot. The peak in the mutual information versus T shows that, while the REM is unlearnable everywhere in the high-temperature regime, it is most unlearnable in an intermediate regime above $T_{c}$, while the entropy is monotonically increasing as a function of T [Fig. 1(b)].

The failure of subextensivity means literally that the influence of one half of this system on the other carries 𝒪(N) bits of information. Intuitively this suggests that we cannot know how one half influences the other unless we specify the state completely. This idea that information is available only once we have access to all the bits in the system reminds us of cryptography, and this connection can be made precise in the context of the random energy model [51].

**FIG. 2.**
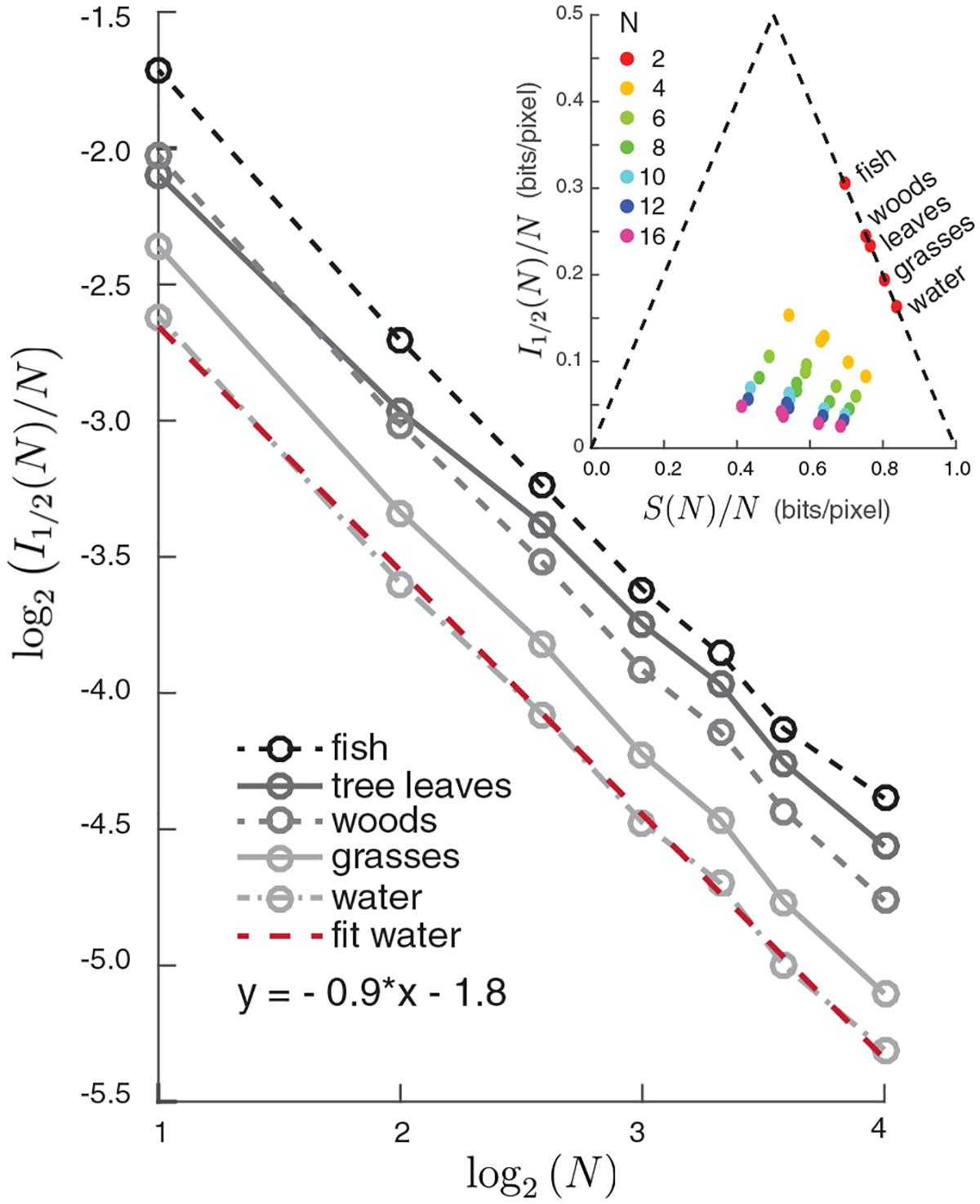
Mutual information between halves of image patches vs patch size in pixels. Data from snapshots out of the Chicago motion database, with different natural environments analyzed separately [52]; error bars at the largest N are a few percent, smaller than the symbols. Inset shows $I_{1/2}(N)$ vs S(N), moving farther away from the bounds as N increases.

As a first example of real-world data, we consider ensembles of images extracted from a large database of natural movies [52]. We discretize to black/white binary pixels with a threshold such that black and white are equally likely. We then analyze contiguous patches of N pixels, and choose halves as the left/right partitions of these patches. With 1200 frames and roughly 200,000 image samples from within these frames, we are able to make reliable estimates of entropy and mutual information out to N~16 pixels; for details see Appendix A. In Fig. 2 (inset) we see that $I_{1/2}(N)$ versus S(N) moves away from the bounds with increasing N, and in the main figure we see explicitly that $I_{1/2}(N)\propto N^{\alpha }$ is strongly subextensive, with α=0.1±0.03, consistently across different natural contexts. This is a striking departure from the behavior of the random energy model, and demonstrates that real-world distributions display subextensivity. Adding noise to these image patches would degrade this feature of the distributions and they would flow toward the I/N=0,S/N=1 lower right corner of the Fig. 2 inset, also losing the scaling we observe with system size.

## III. COMPRESSIBILITY

It is perhaps surprising that real-world data meet the conditions for being well approximated by a model of independent pieces. Still, this is unsatisfying, and we would like to do better. Can we build a model in which the *total* cost ΔL is finite, even as the number of degrees of freedom N becomes large? We will see that this is possible if shared information is compressible.

Let us break the N spins into two groups,

(16) $${\vec{\sigma }}_{K}\equiv \left\{\sigma_{1},\sigma_{2},\ldots ,\sigma_{K}\right\},$$

(17) $${\vec{\sigma }}_{N-K}\equiv \left\{\sigma_{K+1},\sigma_{K+2},\ldots ,\sigma_{N}\right\},$$

with K≪N. The smaller group of K spins could be one of the blocks of size $n_{0}$ from above, but this is not essential. The mutual information

(18) $$I_{0}(N,K)\equiv I\left({\vec{\sigma }}_{K};{\vec{\sigma }}_{N-K}\right)$$

is bounded by S(K) and hence finite, even as N→∞, making it plausible that we do not need to specify all the details of the N-K spins to capture their influence on the K spins. The general idea is to compress our description of ${\vec{\sigma }}_{N-K}$ while maintaining as much information as possible about ${\vec{\sigma }}_{K}$, and this is the information bottleneck problem [53]. Concretely, we map ${\vec{\sigma }}_{N-K}\to X$, maximizing

(19) $$-ℱ=I\left(X;{\vec{\sigma }}_{K}\right)-TI\left(X;{\vec{\sigma }}_{N-K}\right).$$

We can solve this problem with X being a discrete variable of cardinality ‖X‖. As T→0 we recover a deterministic mapping ${\vec{\sigma }}_{N-K}\to X$, and this mapping captures a fraction of the available information,

(20) $$I_{T=0}\left(X;{\vec{\sigma }}_{K}\right)=\left[1-\epsilon_{N}(\|X\|)\right]I_{0}(N,K),$$

where the notation reminds us that the efficiency of capturing information may depend on N.

The intuition of compressibility is that with only $I_{0}$ bits available, we should be able to express the interaction between ${\vec{\sigma }}_{K}$ and ${\vec{\sigma }}_{N-K}$ in roughly $I_{0}$ bits, or in a compressed variable X with ${\text{log}}_{2}\|X\|\sim I_{0}$. To be more precise, let us define a function $F_{N}(\epsilon )$, such that if we compress to within a factor F we capture information to within a factor ϵ,

(21) $${\text{log}}_{2}\|X\|=F_{N}(\epsilon )I_{0}\Rightarrow \epsilon_{N}(\|X\|)=\epsilon .$$

**FIG. 3.**
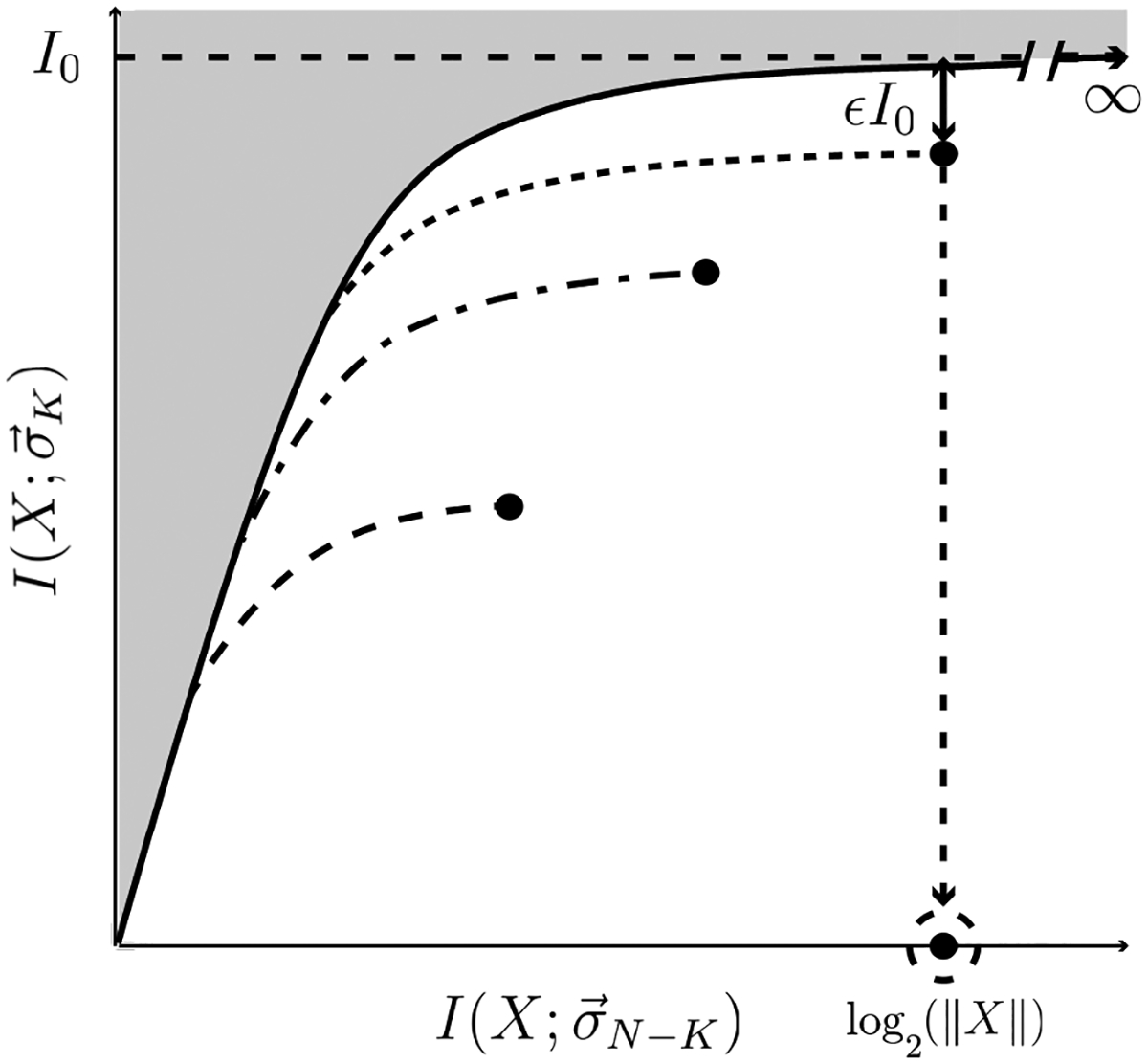
Schematic of the information bottleneck. Solid line divides the forbidden (gray) region from the allowed region. If we solve the bottleneck problem with X as a discrete variable, then at fixed cardinality we vary T in Eq. (19) to trace out the dashed lines, each ending at $I\left(X;{\vec{\sigma }}_{N-K}\right)={\text{log}}_{2}(\|X\|)$. As ‖X‖→∞ we approach saturation, $I\left(X;{\vec{\sigma }}_{K}\right)=I_{0}$, but at finite ‖X‖ we miss by ϵ.

This is illustrated in Fig. 3.

Compression means that we are approximating

(22) $$P\left({\vec{\sigma }}_{K}|{\vec{\sigma }}_{N-K}\right)\approx P\left({\vec{\sigma }}_{K}|X\right).$$

This approximate model has $2^{K}\|X\|$ states, and hence this many parameters. To describe the whole system we need N/K of these models, so the total number of parameters P is given (somewhat generously) by

(23) $${\text{log}}_{2}\;P=K+{\text{log}}_{2}\|X\|+{\text{log}}_{2}(N/K).$$

The cost of coding in this approximate model is the total mutual information we are missing,

(24) $$\Delta L=(N/K)\epsilon_{N}(\|X\|)I_{0}.$$

So to achieve a fixed ΔL at large N, we need to have

(25) $$\epsilon =\frac{K\Delta L}{NI_{0}},$$

which means

(26) $${\text{log}}_{2}\;P=K+F_{N}\left(\epsilon =\frac{K\Delta L}{NI_{0}}\right)I_{0}+{\text{log}}_{2}(N/K).$$

Thus, the number of parameters is set by the behavior of $F_{N}\left(\epsilon =K\Delta L/NI_{0}\right)$ at large N.

The most favorable possibility is that

(27) $$\underset{N\to \infty }{\text{lim}}\;F_{N}(\epsilon =0)=f(K)$$

(28) $$\Rightarrow {\text{log}}_{2}\;P=K+f(K)I_{0}+{\text{log}}_{2}(N/K),$$

and hence P~N. This is what happens in physics problems with local interactions: the impact of the N-K spins on the small region of K spins can be captured by enumerating a fixed number of variables even as N→∞.

The next case is where there is a logarithmic divergence at small ϵ, so that

(29) $$\underset{N\to \infty }{\text{lim}}\;F_{N}\left(\epsilon =\frac{K\Delta L}{NI_{0}}\right)=g(K)\;{\text{log}}_{2}\left(\frac{NI_{0}}{K\Delta L}\right)+\text{const.,}$$

which implies

(30) $${\text{log}}_{2}\;P\sim \left[1+g(K)I_{0}\right]\;{\text{log}}_{2}\;N+\text{const}.$$

Thus, the number of parameters is polynomial in the number of spins, although possibly with a large power.

The logarithmic behavior of $F_{N}\left(\epsilon =K\Delta L/NI_{0}\right)$ as N→∞ is realized in certain models with long-ranged interactions, including mean-field models. This is easiest to see at K=1, where the impact of all N-1 spins on the one spin of interest can always be summarized by an effective field h. As N→∞, this field becomes a continuous variable, chosen from a distribution P(h) which could be different at every spin. Compressing the state of the N-1 spins is equivalent to representing the continuous h by the discrete X; information is lost because there is some range of h values that are assigned to the same X. If ‖X‖ is large and this information loss is small, then we will have $\epsilon \sim {\left\langle (\delta h{)}^{2}\right\rangle }_{X}$, the variance of h at fixed X. Crudely speaking, compression takes the full dynamic range $H_{N}$ of the effective field, which may depend on N, and divides it into ‖X‖ bins, so that

(31) $${\left\langle (\delta h{)}^{2}\right\rangle }_{X}\sim \frac{H_{N}^{2}}{\|X{\|}^{2}},$$

and hence $F_{N}(\epsilon )\sim {\text{log}}_{2}\left(H_{N}^{2}/\epsilon \right)$, so that

(32) $${\text{log}}_{2}\;P\sim {\text{log}}_{2}\left(\frac{NH_{N}^{2}}{\Delta L}\right)+{\text{log}}_{2}(N),$$

where we drop N-independent constants.

As an example, in the disordered phase of a mean-field ferromagnet, we have $H_{N}\sim 1/\sqrt{N}$, which gives a number of parameters again linear in the number of spins. This still seems like too many parameters for a mean-field model, but we have not assumed that all spins are identical, which would take P→P/N. In contrast, if $H_{N}\sim 1$ at large N, we have $P\propto N^{2}$. The last case we might worry about is if the typical field $H_{N}$ grows with N, but then the entropy per spin will vanish as N→∞. Notice that these results, perhaps surprisingly, do not depend on the usual assumption of pairwise interactions, although they show that the number of parameters we need is of the same order as in a pairwise model.

While a logarithmic divergence in $F_{N}(\epsilon )$ leaves us with a polynomial number of parameters, a linear divergence implies that the code words needed to describe the effect of N-K spins on the small cluster of K spins have ~N bits, and no compression is possible. In this case we are back to a number of parameters that is exponential in N. Together, these three examples illustrate three different regimes, from strongly compressible to fully incompressible, showing how compressibility is sufficient to bound the number of parameters P(N,ϵ).

## IV. COMPRESSIBILITY OF NEURAL INTERACTIONS

To see how this works in practice, we look at experiments on the activity of N=160 ganglion cells in the salamander retina as it responds to naturalistic movies [26]. In these data, $\sigma_{\text{i}}=1(0)$ corresponds to the presence (absence) of an action potential from neuron i in a window of duration Δτ=20ms; we note at the outset that these data are not low dimensional [26,54].

**FIG. 4.**
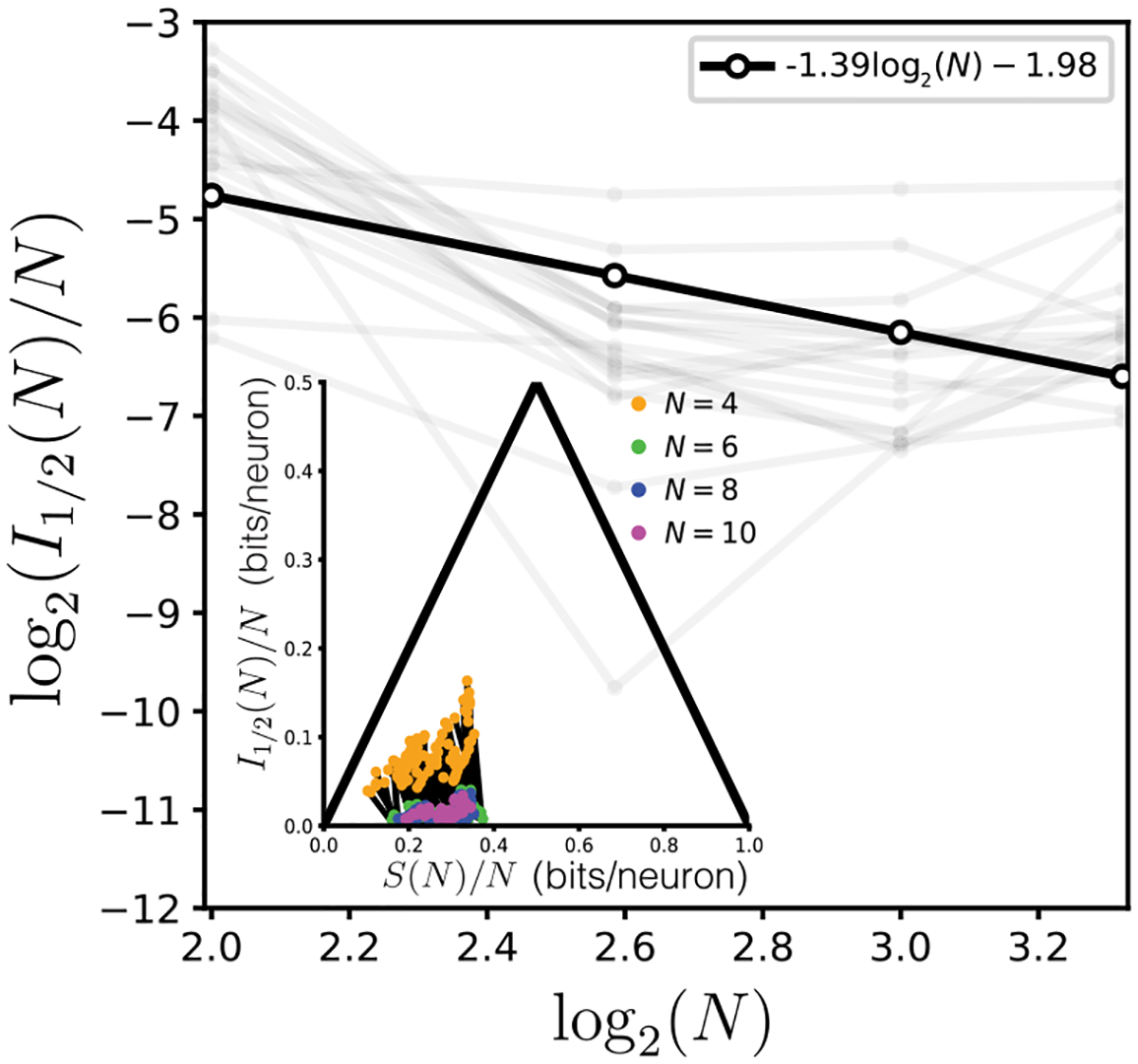
Mutual information between halves of neuron groups vs group size. Gray traces correspond to different groups from the whole population and the black line shows the linear fit over N=160 groups. As explained in the text, groups are formed greedily from the most highly correlated neurons. Inset shows $I_{1/2}(N)$ vs S(N) as in Fig. 2.

We first test for subextensivity of the mutual information. In contrast to systems with local interactions, however, there is no unique way of considering groups of different size. The test will be more compelling if we have groups that share large amounts of information, so we start with one neuron and then add greedily, each time choosing the cell N+1 so that $I\left(\sigma_{N+1};\left\{\sigma_{\text{i}=1,\cdots ,N}\right\}\right)$ is maximized. We can then estimate the mutual information between halves of the group, and these estimates are reliable up to N~10, following the methods of Appendix A; results are shown in Fig. 4. While there is considerable variation across different groups, the mean behavior is $I_{1/2}(N)/N\sim N^{-1.39}$. For all groups of neurons, the trajectory of $I_{1/2}(N)$ versus S(N) moves away from the bounding triangle as we increase N. Together these results suggest that mutual information is strongly subextensive in this system.

To analyze compressibility, we choose one neuron in the population as $\sigma_{0}$, and then order the remaining neurons by their mutual information $I\left(\sigma_{0};\sigma_{\text{i}}\right)$. To be sure that we can calibrate the fraction of information that we capture, we focus on smaller groups of K neurons. Choosing K involves a trade–off between statistical reliability and compression significance: at larger K it is more significant to find a successful compression, but it is more difficult to make reliable statistical inferences. As we will see, K=8 provides an effective compromise (Appendix A). Within the bounds of our ability to make reliable estimates, choosing a different K does not change our main conclusions on the learnability and compressibility of neuronal populations (Appendix B).

The effective interactions between $\sigma_{0}$ and the activity of the other K neurons, $\left\{\sigma_{\text{j}=1,\cdots ,K}\right\}$, are described by the conditional distribution $P\left(\sigma_{0}|\left\{\sigma_{\text{j}}\right\}\right)$, and we can always write this as an effective field $h_{\text{eff}}\left(\left\{\sigma_{\text{j}}\right\}\right)$ acting on $\sigma_{0}$,

(33) $$P\left(\sigma_{0}|\left\{\sigma_{\text{j}}\right\}\right)=\frac{1}{Z\left(\left\{\sigma_{\text{j}}\right\}\right)}\text{exp}\left[\sigma_{0}h_{\text{eff}}\left(\left\{\sigma_{\text{j}}\right\}\right)\right].$$

If we can understand this distribution for each possible choice of $\sigma_{0}$, then we will have understood the whole network.

With K neurons in the set $\left\{\sigma_{\text{j}}\right\}$, then in principle we need $2^{K}$ different values of the effective field

(34) $$h_{\text{eff}}\left(\left\{\sigma_{\text{j}}\right\}\right)=\text{ln}\left[\frac{P\left(\sigma_{0}=1|\left\{\sigma_{\text{j}}\right\}\right)}{P\left(\sigma_{0}=0|\left\{\sigma_{\text{j}}\right\}\right)}\right].$$

A conventional simplification is to expand $h_{\text{eff}}$ in a series,

(35) $$h_{\text{eff}}\left(\left\{\sigma_{\text{j}}\right\}\right)=h_{0}+\sum_{\text{j}}J_{0\text{j}}^{\left(2\right)}\sigma_{\text{j}}+\frac{1}{2}\sum_{\text{j},\text{k}}J_{0\text{jk}}^{\left(3\right)}\sigma_{\text{j}}\sigma_{\text{k}}+\cdots .$$

Stopping with the second term corresponds to allowing only pairwise interactions in the effective Hamiltonian H=-lnP, and gives a description of $P\left(\sigma_{0}|\left\{\sigma_{\text{j}}\right\}\right)$ with K+1 rather than $2^{K}$ parameters.

**FIG. 5.**
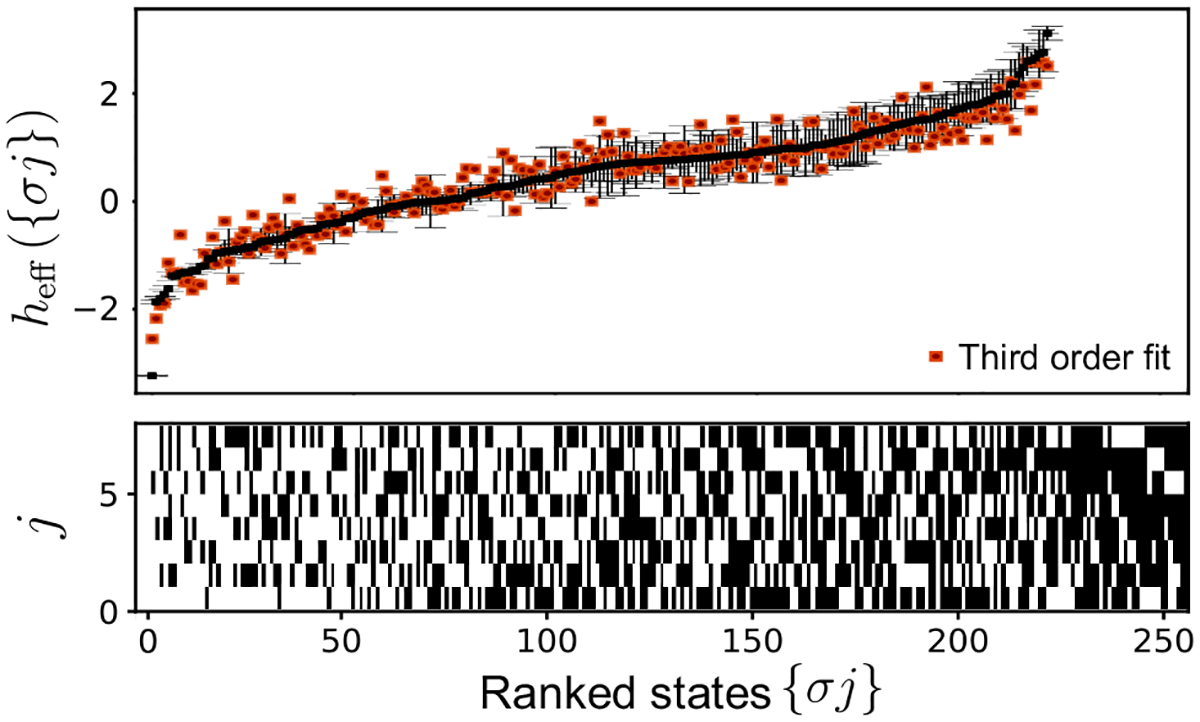
Effective field $h_{\text{eff}}\left(\left\{\sigma_{\text{j}}\right\}\right)$ as a function of the states $\left\{\sigma_{\text{j}}\right\}$, in rank order. Mean, with error bars estimated from the standard deviation across random halves of data (black), and best least squares fit to Eq. (35) truncated at third order (orange). Ranked states are represented in the figure below the trace, with black indicating that a neuron is “on,” $\sigma_{\text{j}}=1$. States at far right are not observed in data.

Consider the K=8 neurons that share the most information with some particular $\sigma_{0}$. Figure 5 shows the effective field $h_{\text{eff}}$ as function of the state $S=\left\{\sigma_{\text{j}}\right\}$ for this example. We note that 218 of the 256 possible states S are visible in the data. If we try to describe these data through Eq. (35), then even including terms up to $J^{\left(3\right)}$ leaves small but significant scatter beyond the measurement errors. This is a very explicit way of seeing that stopping with $J^{\left(2\right)}$, and using a pairwise Ising model, misses significant parts of the underlying correlation structure in this system [26,36]. However, we hope not to need all the possible terms out to $J^{\left(8\right)}$. Can we show that interactions are compressible, and this captures the dependences with fewer parameters?

Compressibility means that we do not need to keep every detail of the network state $\left\{\sigma_{\text{j}}\right\}$ to make reliable predictions of the effective field. Concretely, this means compressing $\left\{\sigma_{\text{j}}\right\}\to \tilde{\sigma }$, where $\tilde{\sigma }$ takes on M states, with $M\ll 2^{K}$; we have changed notation from X to $\tilde{\sigma }$ to emphasize that we’re constructing coarse-grained or compressed descriptions of the variables σ. As before we are interested in the information that these compressed variables share with $\sigma_{0}$, so we want to choose the mapping $\left\{\sigma_{\text{j}}\right\}\to \tilde{\sigma }$ that maximizes $I\left(\tilde{\sigma };\sigma_{0}\right)$, and we write this maximum as $I_{\text{max}}(M)$. If we can achieve $\text{FI}=I_{\text{max}}(M)/I\left(\sigma_{0};\left\{\sigma_{\text{j}}\right\}\right)\approx 1$ for small M even at large K, then we have tamed the combinatorial explosion.

We consider here only deterministic mappings $\left\{\sigma_{\text{j}}\right\}\to \tilde{\sigma }$, which means that we are solving the zero temperature or hard clustering limit of the information bottleneck problem Eq. (19) [53,55],

(36) $$\underset{\left\{\sigma_{\text{j}}\right\}\to \tilde{\sigma }}{\text{max}}\;I\left(\tilde{\sigma };\sigma_{0}\right),\;\|\tilde{\sigma }\|=M.$$

A simple algorithm for solving this problem is to start with some random assignment $\left\{\sigma_{\text{j}}\right\}\to \tilde{\sigma }$, then compute

(37) $$P\left(\sigma_{0};\tilde{\sigma }\right)=\sum_{\left\{\sigma_{\text{j}}\right\}\in \tilde{\sigma }}P\left(\sigma_{0}|\left\{\sigma_{\text{j}}\right\}\right)P\left(\left\{\sigma_{\text{j}}\right\}\right),$$

(38) $$P(\tilde{\sigma })=\sum_{\left\{\sigma_{\text{j}}\right\}\in \tilde{\sigma }}P\left(\left\{\sigma_{\text{j}}\right\}\right),$$

with $P\left(\sigma_{0}|\tilde{\sigma }\right)=P\left(\sigma_{0};\tilde{\sigma }\right)/P(\tilde{\sigma })$ as usual. We then reassign each particular state $\left\{\sigma_{\text{j}}\right\}$ to the compressed variable by minimizing the Kullback-Leibler divergence,

(39) $$\left\{\sigma_{\text{j}}\right\}\to \text{arg}\;\underset{\tilde{\sigma }}{\text{min}}\;\sum P\left(\sigma_{0}|\left\{\sigma_{\text{j}}\right\}\right)\;{\text{log}}_{2}\left[\frac{P\left(\sigma_{0}|\left\{\sigma_{\text{j}}\right\}\right)}{P\left(\sigma_{0}|\tilde{\sigma }\right)}\right].$$

Iterating, we arrive at a mapping $\left\{\sigma_{\text{j}}\right\}\to \tilde{\sigma }$ that maximizes $I\left(\sigma_{0};\tilde{\sigma }\right)$.

**FIG. 6.**
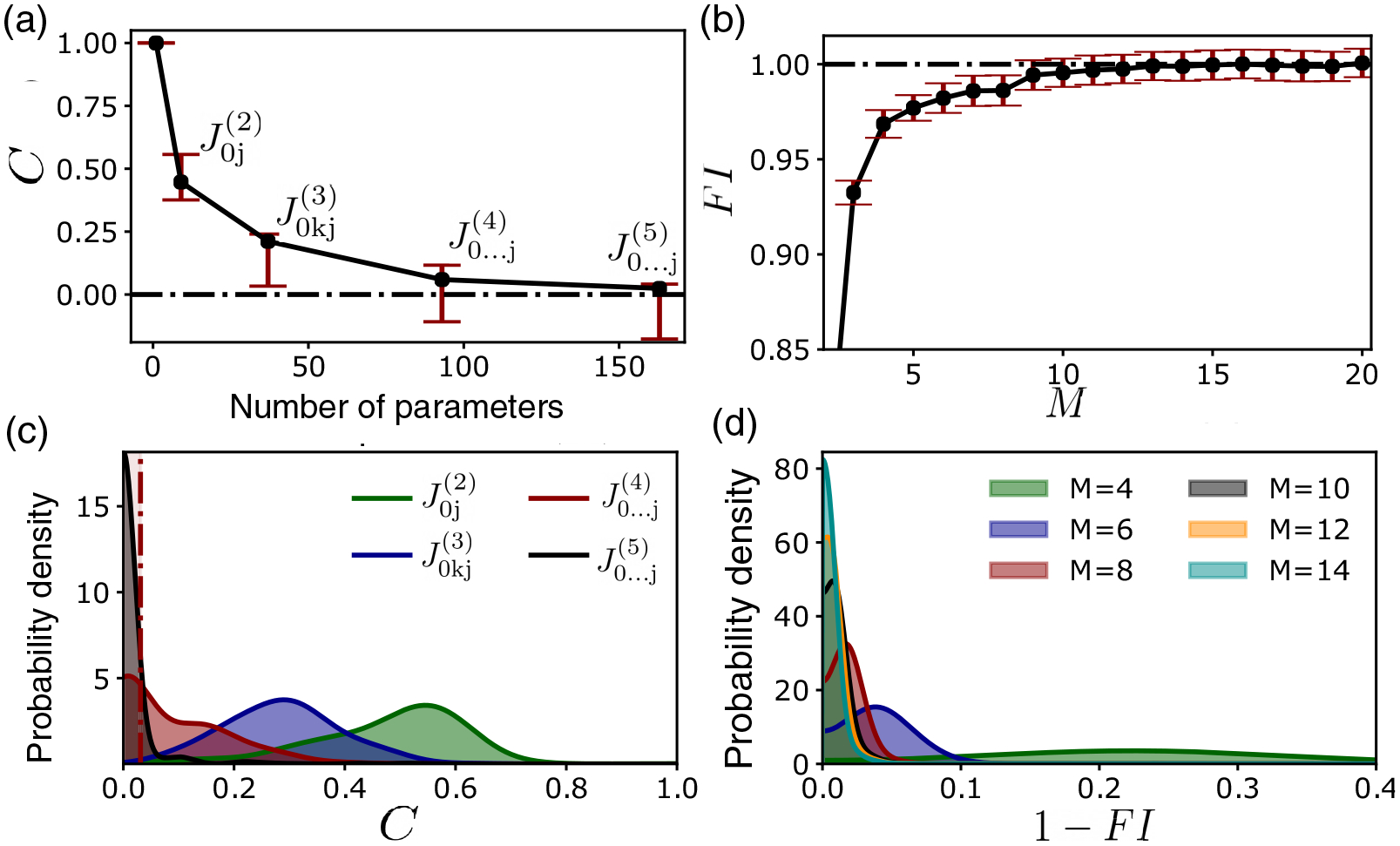
Series expansions vs compression. (a) Coding cost [Eq. (41)] as a function of the number of parameters for the series expansion in Eq. (35). Black points from analysis with all data, error bars are standard deviation across random choices of learning from 60% of the data and testing on the remaining 40%. (b) Fraction of mutual information captured as a function of the number of states M in the compressed representation ${\tilde{\sigma }}_{i}$. Error bars from analyses of random subsets of the data. (c) Coding cost probability density over all possible choices of $\sigma_{0}$. Each curve correspond to a different order truncation, $J_{0j}^{\left(i\right)}$, of the series expansion [Eq.(35)]. (d) Probability density of the fractional information over all possible choices of $\sigma_{0}$. Each curve correspond to a different value of M. We used a weighted-KDE method for the inference of the probability densities, considering the measured error bars of each choice of $\sigma_{0}$.

To compare the performance of compression methods with the performance of series expansions, we use once more the idea that approximations to the true probability distribution define suboptimal codes, and the excess code length measures the cost of the approximation. In this case we are building a code for the binary variable $\sigma_{0}$ conditional on the state of the other K neurons; the optimal code length is $L_{\text{min}}$. If we have an approximate model for $h_{\text{eff}}\left(\left\{\sigma_{\text{j}}\right\}\right)\approx {\overset{ˆ}{h}}_{\text{eff}}\left(\left\{\sigma_{\text{j}}\right\}\right)$, then the mean code length is

(40) $$L_{\text{approx}}=-\frac{1}{\text{ln}\;2}\left\langle \sigma_{0}{\overset{ˆ}{h}}_{\text{eff}}\left(\left\{\sigma_{\text{j}}\right\}\right)\right\rangle +\left\langle {\text{log}}_{2}\left[1+e^{{\overset{ˆ}{h}}_{\text{eff}}\left(\left\{\sigma_{\text{j}}\right\}\right)}\right]\right\rangle ,$$

where ⟨⋯⟩ is an average over the observed states of the system. The most naive approximation ignores interactions, assigning the same effective field to all states, and this “independent” code has length $L_{\text{ind}}$. A natural measure of coding cost is then

(41) $$C=\left(L_{\text{approx}}-L_{\text{min}}\right)/\left(L_{\text{ind}}-L_{\text{min}}\right),$$

which ranges between zero and one. For the case where we do a proper compression $\left\{\sigma_{\text{j}}\right\}\to \tilde{\sigma }$, then C=1-FI, where FI is the fraction of the mutual information that we capture (see above), but the coding cost is defined more generally, e.g., in the truncated series expansions of Eq. (35). In Figs. 6(a) and 6(b) we compare the optimal compressions with the series expansion, and find that compression into the best choice of M~10 states performs as well as including 163 parameters to describe 5th order interactions.

To check that these results do not depend on our choice of the central neuron $\sigma_{0}$, in Figs. 6(c) and 6(d) we show the distribution of coding cost and fractional information across these choices. We see in Fig. 6(c) that even getting within ten percent of the optimum across the majority of neurons requires extending the series expansion to fifth order, that is including terms up to $J^{\left(5\right)}$ in Eq. (35). While for specific choices of $\sigma_{0}$, making truncations at lower orders might lead to reasonable descriptions of the system, we find that this is not the rule at the population level. In contrast, Fig. 6(d) shows that by compressing into M~11-15 states we achieve a code that captures all but a few percent of the available mutual information, for all neurons in the population. To summarize, in this network we can describe the influence of K=8 neurons on one neuron using just M=11-15 parameters, but this most efficient description does not correspond to a simple choice of pairwise or other low-order interactions.

**FIG. 7.**
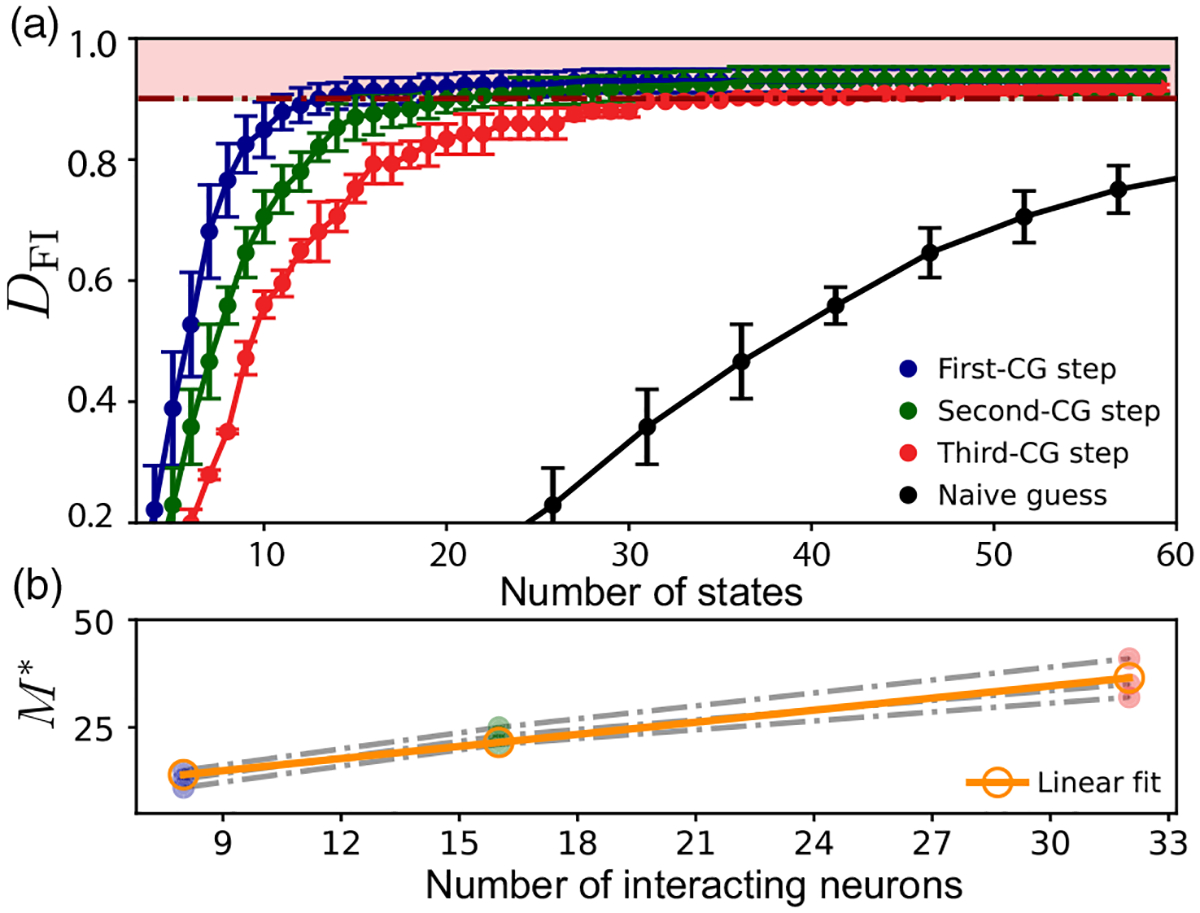
Capturing mutual information with a limited number of states. (a) Fraction of cells $\sigma_{0}$ and groups $\left\{\sigma_{\text{j}}\right\}$ such that compression into M states captures the available mutual information, within error bars. Successive coarse-graining steps as described in the text. We define $M^{*}$ as the minimum number of states needed to achieve complete compression in 90% of the cases. The black curve corresponds to the naive expectation for for the third step of coarse-graining if the number of states we need grows exponentially with the number of neurons. (b) Minimum number of states $M^{*}$ as a function of the coarse-graining step. Dashed curves (gray) correspond to different compression iterations, which vary because of noise in our estimates. For comparison, a linear relation is shown in orange.

Estimates of mutual information come with errors, and so statements about the number of states needed to capture a given fraction of the information also have uncertainty (Appendix A). For each choice of $\sigma_{0}$ and $\left\{\sigma_{\text{j}}\right\}$ out of the network, estimates of FI are accompanied by an error $\Delta_{\text{FI}}\left(\sigma_{0},\left\{\sigma_{\text{j}}\right\}\right)$, and as a global measure $\Delta_{\text{FI}}$ we take the median of these errors. If we choose a fixed number of states M for the compression, then across all choices of $\sigma_{0}$ and $\left\{\sigma_{\text{j}}\right\}$ we will find a fraction $D_{\text{FI}}$ for which the estimate of FI is larger than $1-\Delta_{\text{FI}}$, i.e., the information captured is within errors of the information available. Figure 7(a) shows the dependence of $D_{\text{FI}}$ on the number of states M, and we see that in 90% of all the relevant groups we achieve essentially perfect compression with $M^{*}\sim 11$ states. We can do this analysis not just for interactions between a single cell $\sigma_{0}$ and its K most informative partners, $S_{1}=\left\{\sigma_{1},\ldots ,\sigma_{8}\right\}$, but also for interactions with successively less informative groups $S_{n}=\left\{\sigma_{k(n-1)+1},\ldots ,\sigma_{kn}\right\}$, and the result are the same up to n=8. Note that with n=8 we are covering 0.4N of the cells in the entire population, and that $I\left(\sigma_{0};S_{n}\right)$ is within error bars of zero for n>8.

## V. ITERATED COMPRESSION

The compression $\left\{\sigma_{\text{j}}\right\}\to \tilde{\sigma }$ is reminiscent of the block spin construction in the renormalization group (RG) [56,57]. We recall that block spins are coarse-grained variables that replace groups of spins. In the present context, it is important to remember that coarse-graining can be thought of as data compression, and vice versa. By analogy with the RG, then, we would like to do iterative compression.

Concretely, we are focused on a variable $\sigma_{0}$ and have ordered the remaining variables $\sigma_{\text{j}}$ by their mutual information with $\sigma_{0}$. Our first coarse-graining step has been to take these variables in groups of K=8, and compress according to the solution of the optimization problem in Eq. (36), which gives us

(42) $$\left\{\sigma_{1},\sigma_{2},\cdots ,\sigma_{8}\right\}\to {\tilde{\sigma }}_{1}^{\left(1\right)}\;\left\{\sigma_{9},\sigma_{10},\cdots ,\sigma_{16}\right\}\to {\tilde{\sigma }}_{2}^{\left(1\right)}\;\cdots ,$$

where each of the variables ${\tilde{\sigma }}_{1}^{\left(1\right)}$ has M states and the superscript reminds us that this is only the first step of coarsegraining. To iterate, we take pairs of these variables and compress again, e.g.,

(43) $$\left({\tilde{\sigma }}_{1}^{\left(1\right)},{\tilde{\sigma }}_{2}^{\left(1\right)}\right)\to {\tilde{\sigma }}_{1}^{\left(2\right)},$$

where again the mapping is chosen to maximize the mutual information $I\left(\sigma_{0};{\tilde{\sigma }}_{1}^{\left(2\right)}\right)$. We can keep iterating,

(44) $$\left({\tilde{\sigma }}_{1}^{\left(2\right)},{\tilde{\sigma }}_{2}^{\left(2\right)}\right)\to {\tilde{\sigma }}_{1}^{\left(3\right)},$$

always with the same principle of choosing the compression that maximizes the mutual information with $\sigma_{0}$. Note that in our initial compression $\left\{\sigma_{\text{j}}\right\}\to {\tilde{\sigma }}^{\left(1\right)}$, we chose K=8 cells and hence 256 states. In the second step, Eq. (43), we start with $M^{2}\sim 256$ states again, which means that we have the same high level of control over sampling problems. At the third step, Eq. (44), we start with somewhat more states, but still sampling is under control, and successive coarse-graining steps are entropically comparable.

It is not surprising that successive stages of compression or coarse-graining require more states to capture all the available mutual information [Fig. 7(a)]. What is surprising is that the minimal number of states $M^{*}$ seems to grow linearly rather than exponentially as we proceed through multiple stages, as seen in Fig. 7(b). After three stages, we are describing the interactions of $\sigma_{0}$ with 32 other cells using only $M_{3}^{*}=32$ states.

If we did not know about subextensivity we might naively expect that to capture information from a group of N neurons requires $M\sim 2^{\gamma N}$ states, or more generally that our success in capturing information should depend on M and N as $D_{\text{FI}}(M,N)=f\left(M/e^{\gamma N}\right)$. An estimate of γ comes from $M_{2}^{*}/M_{1}^{*}=e^{\gamma \left(N_{2}-N_{1}\right)}$, where $N_{1}=8$ and $N_{2}=16$ are the sizes of the group at the first steps of coarse-graining, so that γ=0.1026. We then can use this to predict $D_{\text{FI}}$ at the third stage of coarse-graining as a stretched version of the curve at the second stage (black curve in Fig. 7), where the stretching factor along the M axis is $e^{\gamma \left(N_{3}-N_{2}\right)}$, with $N_{3}=32$. We see that the real data are dramatically different.

The linear growth of $M^{*}$ with the number of neurons is explicit evidence that we have tamed the combinatorial explosion, combining the compressibility of interactions with an RG–inspired iteration scheme. The scaling of $M^{*}$ is what we might expect in a model with pairwise interactions, or if single neurons coupled only to the total activity of other neurons, but neither of these simplifications is correct.

As a further test of these ideas we have looked at experiments on a very different network of neurons, in the mouse hippocampus [27,30]. The results, described in Appendix B, are very much the same, but perhaps less surprising since maximum entropy models with only pairwise interactions already provide an excellent description of these data, matching the higher order correlations within experimental error [27,36]. In contrast, as emphasized in Ref. [26], for the population of cells in the retina the pairwise models show small but significant deviations from the data, and this has led to the exploration of several alternatives [26,33,34].

Where previous work has focused on simple forms of the interactions, taking intuition both from physics and from neurobiology, the point of our discussion is not to identify the correct model, but to understand why *any* simple model can succeed. Indeed, the results of this approach in two very different neuronal populations suggest that compressibility is a more general and intrinsic property of large neuronal networks.

## VI. IMPLICATIONS FOR THE NEURAL CODE

We have emphasized that compressibility allows us to give a simpler description of correlation structure in the patterns of activity seen in populations of retinal ganglion cells. It is important that compressibility also has implications for the functional behavior of the network as it encodes the visual world.

The fact that we can compress the interactions means that we have a good estimate of the information that each neuron shares with the rest of the network, $I\left(\sigma_{0};\tilde{\sigma }\right)$. But what is this information? A natural guess is that information shared among visual neurons is information about the visual world. To test this, we note that the experiments in Ref. [26] include many repeated presentations of the same movie. This repetition means we can estimate the distribution of neural activity conditional on the visual stimulus s, and consequently the information that neurons carry about the stimulus, $I\left(\sigma_{0};s\right)$.

Information estimates would be difficult if we were to ask about the patterns of activity across many cells. But if we ask about just one cell there is more than enough data in these experiments to reach reliable conclusions about the information that this cell’s response carries about the visual input [58,59]. Importantly, these information estimates $I\left(\sigma_{0};s\right)$ are independent of model assumptions about which features of the visual stimulus are being represented and independent of what is happening in the other neurons of the network. Thus, while we can think $I\left(\sigma_{0};\tilde{\sigma }\right)$ as a measure of population-level correlations, $I\left(\sigma_{0};s\right)$ is an independent measure of visual information.

**FIG. 8.**
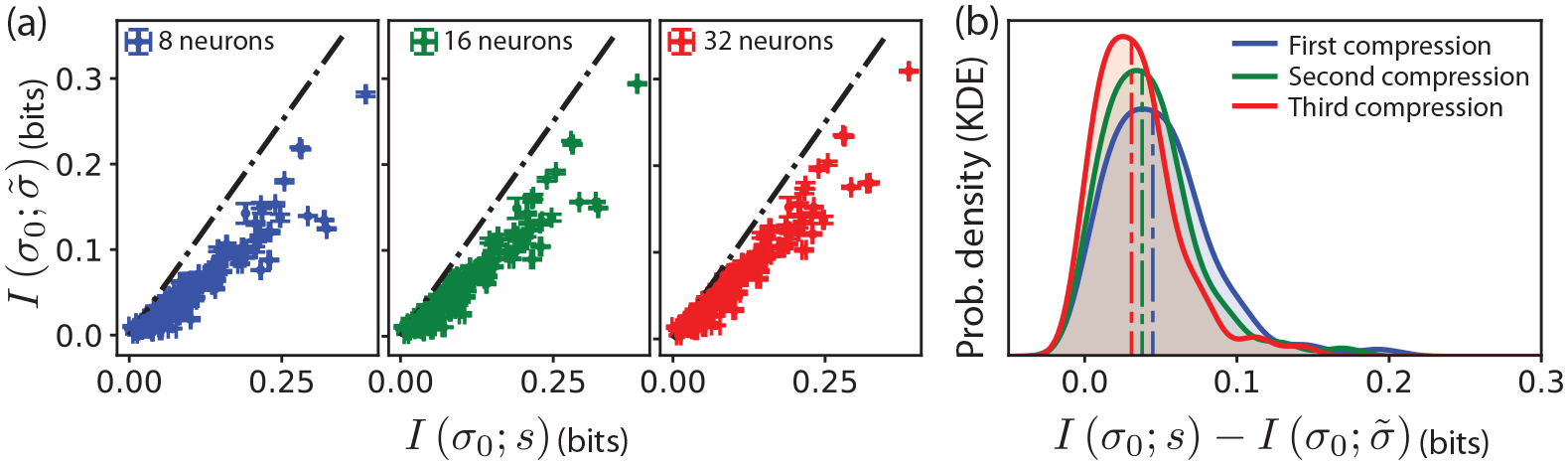
Shared information vs visual information. a) Information that a single neuron shares with the network vs information that this neuron conveys about the stimulus. Plot corresponds to the first (blue, 8 neurons), second (green, 16 neurons) and third (red, 32 neurons) coarse-graining steps. Estimates and errors as in Ref. [58].) Kernel density estimator (KDE) of the difference between the information shared with the stimulus vs information about the network for each step. Dashed lines correspond to the medians.

Figure 8 shows the information that each single cell carries about the visual stimulus, $I\left(\sigma_{0};s\right)$, compared with the information that this single neuron shares with the network, $I\left(\sigma_{0};\tilde{\sigma }\right)$. We see that the intuition connecting shared information with visual information is surprisingly accurate. In fact, as we consider larger groups of neurons, the shared information approaches the visual information more and more closely, within (small) error bars. This equality has a surprising consequence.

Suppose that we ask not about the visual information carried by a single neuron, but rather about the *extra* information that this neuron carries beyond what all the other neurons tell the brain about the visual inputs. Formally, this extra information is

(45) $$\Delta I\left(\sigma_{0};s\right)=I\left(\left\{\sigma_{\text{j}}\right\},\sigma_{0};s\right)-I\left(\left\{\sigma_{\text{j}}\right\};s\right).$$

Following arguments about synergy and redundancy among different components of the neural response [59], we can rewrite the extra information in terms of shared information (see Appendix C for details):

(46) $$\Delta I\left(\sigma_{0};s\right)=I\left(\sigma_{0};s\right)+I\left(\sigma_{0};\left\{\sigma_{\text{j}}\right\}|s\right)-I\left(\sigma_{0};\left\{\sigma_{\text{j}}\right\}\right).$$

In this expression, $I\left(\sigma_{0};s\right)$ is the information that a single neuron carries about the visual stimulus and $I\left(\sigma_{0};\left\{\sigma_{\text{j}}\right\}\right)$ is the information that it shares with the network, as before, while $I\left(\sigma_{0};\left\{\sigma_{\text{j}}\right\}|s\right)$ is the (average) mutual information between $\sigma_{0}$ and the network given that visual stimulus is known.

The fact that we achieve effective compression of the shared information means we can replace $I\left(\sigma_{0};\left\{\sigma_{\text{j}}\right\}\right)$ by $I\left(\sigma_{0};\tilde{\sigma }\right)$. But then Fig. 8 tells us that $I\left(\sigma_{0};\tilde{\sigma }\right)\approx I\left(\sigma_{0};s\right)$, so that the first and last terms in Eq. (46) cancel, and we are left with

(47) $$\Delta I\left(\sigma_{0};s\right)\approx I\left(\sigma_{0};\left\{\sigma_{\text{j}}\right\}|s\right).$$

This tells us that the retina is operating in a regime where the extra information provided by a single neuron is nearly equal to the information that this neuron shares with the network given that we know the visual stimulus.

A popular model for retinal coding is that individual neurons respond independently to the visual inputs, so that all correlations are inherited from the stimulus;^2^ formally this conditionally independent model is defined by

(48) $$P\left(\left\{\sigma_{\text{j}}\right\}|s\right)=\prod_{\text{j}}Q_{\text{j}}\left(\sigma_{\text{j}}|s\right).$$

If this is true, then $I\left(\sigma_{0};\left\{\sigma_{\text{j}}\right\}|s\right)=0$ and the neuron at the center of our analysis would be completely redundant with the other K neurons, $\Delta I\left(\sigma_{0};s\right)\approx 0$. Stated in a more positive way, the global correlation structure of the retinal population is such that the extra information carried by individual neurons depends entirely on their departure from conditional independence.

Correlations between neurons that persist even when the stimulus is fixed are sometimes called “noise correlations” [61–68]. There is ample experimental evidence for these correlations among retinal ganglion cells, as reviewed for example in Ref. [54], and there are clear mechanisms that could generate such correlations, including the flow of noise from the receptor cells through the retinal circuitry [69]. Nonetheless noise correlations continue to be seen as second order effects, and the conditions under which these correlations can enhance the information content or efficiency of the neural code seem exotic. It thus comes as surprise that the retina is operating in a limit where the information carried by noise correlations is essentially *equal* to the incremental information contributed by a single neuron.

## VII. DISCUSSION

To summarize, the consistent subextensivity of mutual information is a determinant of learnability that makes possible approximate models with a number of parameters that is linear in the number of degrees of freedom while suffering a cost per degree of freedom that vanishes in the thermodynamic limit, and compressibility of the mutual information makes it possible to have only finite total cost in this limit. These results suggest, strongly, that complexity can be tamed without making assumptions about the nature of interactions, generalizing our intuition from physics problems that we understand. Perhaps this also provides new perspectives on why simple models work in the traditional problems of statistical physics.

It is important that the ideas of consistent subextensivity and compressibility apply to real data. We are especially struck by the fact that neural activity in the retina obeys these conditions, providing a basis to understand why the neural code is learnable [33]. As a consequence of learnability, we can perform an iterative, RG-like compression of the effective interactions between neurons that leads to a description in which the influence of N neurons on one central neuron is described by ~N parameters, even though these interactions are not pairwise. This tells us that the collective nature of the retinal code [70] does not require us to learn higher-order interactions order by order. More work is required to exploit this observation in constructing a full model for the joint distribution of activity across a large network, but our results show that such simplified models are possible with essentially zero information loss.

This compressibility has another intriguing consequence in the context of population coding in the brain. Almost from the start of simultaneous recordings from multiple neurons there has been an attempt to separate “signal” and “noise” correlations, often in the hope that noise correlations are small and could be neglected [60,63,64]. While there are examples where noise correlations may have a structure that enhances neural coding [67,68,71,72], we are not aware of an example in which these correlations almost perfectly cancel the redundancy between individual neurons and the larger population, which is what we find here.

The near perfect compressibility of the effective interactions found here tames the combinatorial explosion naïvely expected for collective codes. Exploiting this result we find that the visual information that one cell’s response adds to the information carried by N other cells in the network is essentially equal to the natural measure of noise correlation in bits. In this sense, the system operates in a regime where noise correlations are essential for coding.

We have restricted ourselves to samples from visual scenes and neural responses at one moment in time, but we could generalize to sequences of states over time. The natural division of a trajectory into halves would be to look at the past and future relative to some midpoint, and thus the analog of $I_{1/2}$ is the mutual information between past and future or, more simply, the predictive information in the time series. Because interactions do not extend over arbitrarily long temporal scales, subextensivity of the predictive information is automatic [73], and this subextensivity is what guarantees the convergence to a well-defined entropy rate for the trajectory. There has been interest in analyzing predictive information both for movies [74] and in the responses of the retinal neurons to such inputs (as considered here) [75–78]. A discussion of learnability for models of these time series would add another layer to the broader effort to understand the learnability of natural distributions, and should connect to current excitement about large language models.

We have phrased the problem of understanding a network of neurons as being able to write a good approximation for the joint distribution of activity across the whole population, essentially being able to predict the likelihood of seeing any of the $2^{N}$ combinations of spiking and silence in the network [36]. The motivation is the analogy to equilibrium statistical mechanics, where being able to write the Boltzmann distribution gives us a starting point for calculating all static physical properties of the system. Our exploration of the compressibility of interactions in the network gives us a clear answer to the question of why such simplified models exist, and a surprisingly direct path to conclusions about the function of the retina as an encoder of the visual world.

## Figures and Tables

**FIG. 9.**
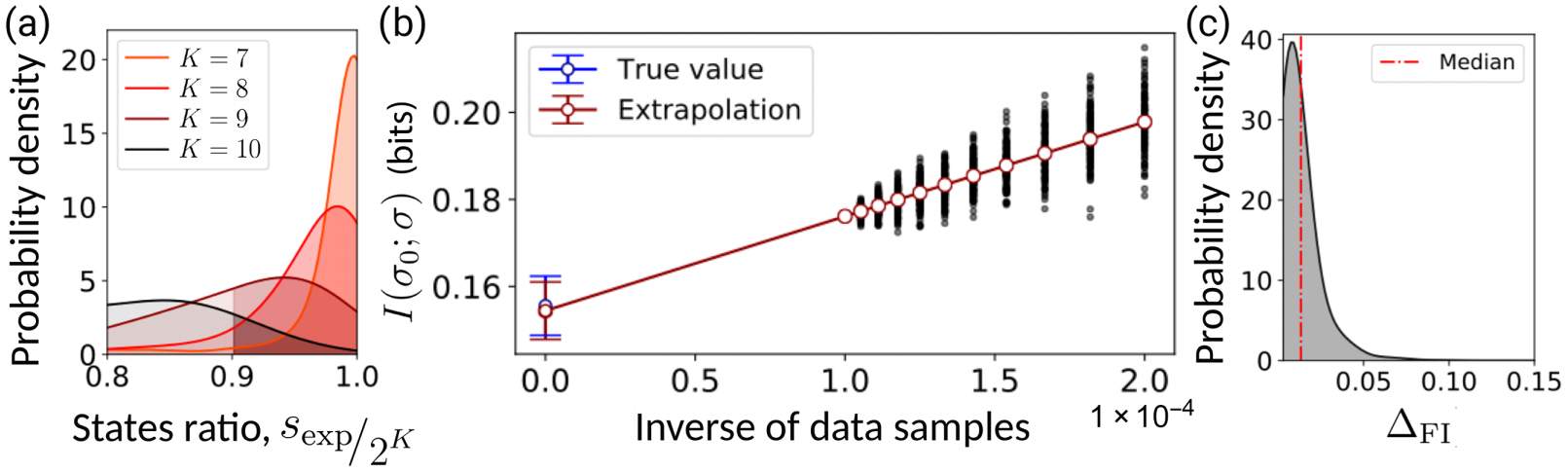
(a) Probability density for the fraction of possible states $\left\{\sigma_{\text{j}}\right\}$ that we observe in the data, for different choices of the neighborhood size K. The state ratio is defined as the number of states $S_{\text{exp}}$ that we find relative to the possible number $2^{K}$. The distribution is across many randomly chosen groups of K neurons from the full population of N=160 cells. (b) Estimates of mutual information as a function of the (inverse) number of samples for the Zipf-like model described in the text. Examples (black), means (red circles), linear fit (red line) and extrapolation with errors; exact result shown for comparison with expected estimation errors (blue). (c) Probability distribution of the fractional information error across all $\sigma_{0}$.

**FIG. 10.**
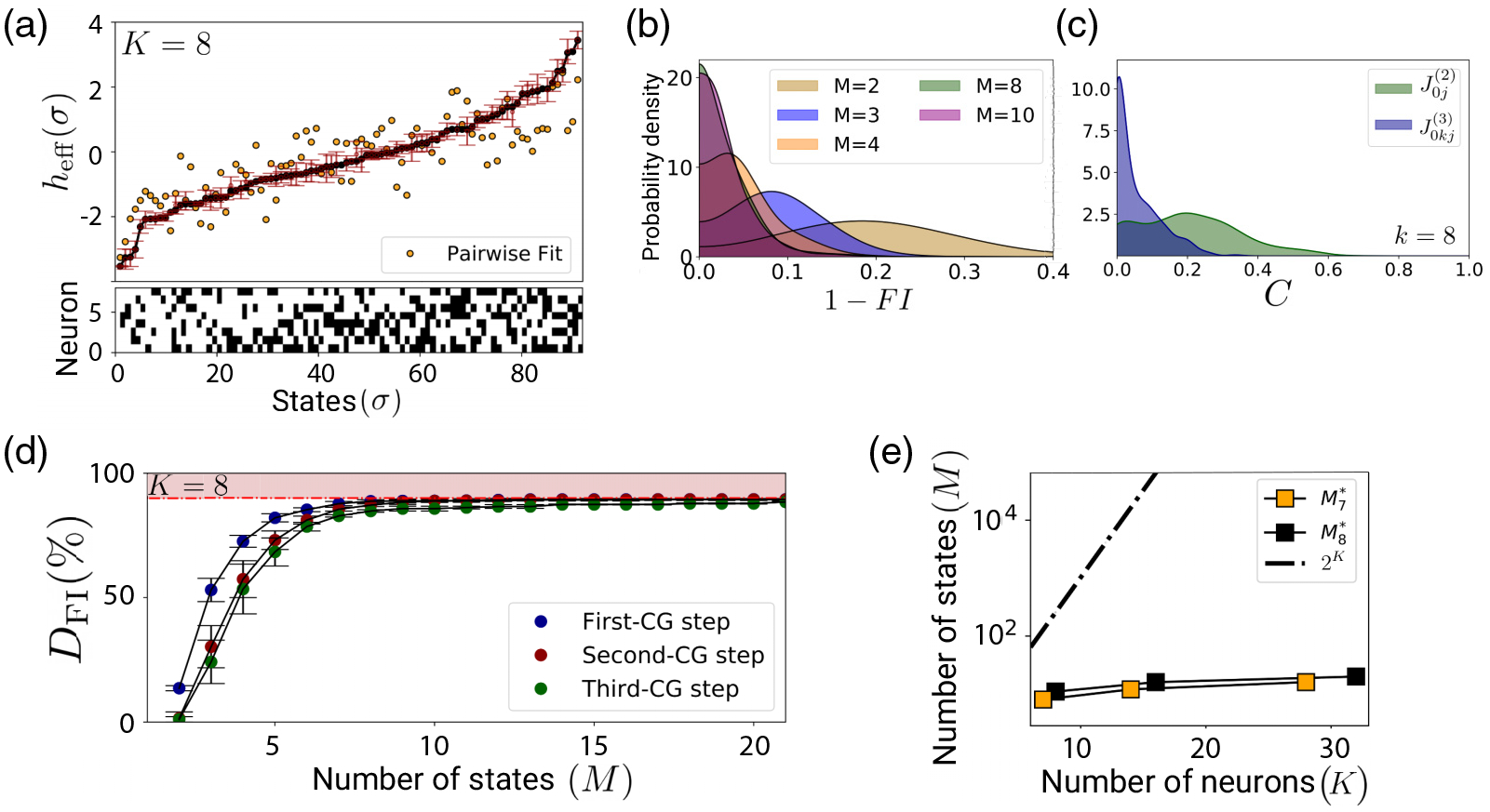
Compressibility in a hippocampal population. (a) The effective field $h_{\text{eff}}(\sigma )$ as a function of the states in σ, in rank order for K=8. Mean, with error bars estimated from the standard deviation across random halves of the data (black) and best least squares fit to Eq. (35) truncated at third order (orange). Ranked states are represented in the figure below the trace, with black indicating that a neuron is active, $\sigma_{\text{j}}=1$. Only experimentally observed states are shown. (b) Probability density of the fractional information over all possible choices of $\sigma_{0}$. Each curve correspond to a different value of M. We used a weighted-KDE method for the inference of the probability densities, considering the measured error bars at each choice of $\sigma_{0}$. (c) Probability distribution of coding costs across all choices of $\sigma_{0}$. Each curve correspond to a different order truncation, $J_{0i}^{\left(\ell \right)}$, of the series expansion. (d) Fraction of cells $\sigma_{0}$ and groups $\sigma^{\left(\ell \right)}$ such that compression into M states captures the available mutual information, within error bars for K=8. Colors represent successive coarse-graining steps as described in the text. We define $M^{*}$ as the minimum number of states needed to achieve complete compression in 90% of the cases. (e) Number of states as a function of the number of neurons for K=7 (orange markers) and K=8 (black markers). The dashed line shows the possible number of states $2^{K}$.

## Data Availability

The data that support the findings of this article are openly available [79].

## Footnotes

1 Our notion of learnability here is a bit different from the usual. In traditional learning theory, one asks for the number of samples required to identify, within some error bounds, the best model in a given class, and the class is defined by the number of parameters or some other complexity measure [39]. Here we are interested in the absolute performance of models, and ask about the number of parameters needed to reach a fixed criterion as the number of degrees of freedom in the system changes. We take as given that the number of needed samples will scale with the complexity of models, and sometimes interchange these quantities, colloquially; our focus is on their scaling with system size.

2 This idea has many origins; it has been used as a simplifying hypothesis but also as a conjectured principle. A particularly strong form of the idea is presented in Ref. [60].

## APPENDIX A: ESTIMATION OF MUTUAL AND FRACTIONAL INFORMATION FOR FINITE DATA

Our strategy for estimating information theoretic quantities follows that used in Ref. [58]: we vary the number of samples that we use in making our estimates, verify that we are in the regime where sample size dependence is as expected from perturbation theory, and extrapolate to infinite data. This approach has a long history, and is reasonably well known; a review can be found in Sec. A.8 of Ref. [80]. We give some details here, in the hope of making the discussion more accessible.

Estimating the mutual information between a single neuron, $\sigma_{0}$, and a group of K neurons, $\sigma \equiv \left\{\sigma_{\text{j}=1,\cdots ,K}\right\}$, requires inferring the corresponding $2^{K+1}$ state probabilities. To begin, as shown in Fig. 9(a), the choice of K=8 allows the observation of >90% of the states for almost all possible choices of $\sigma_{0}$. We then choose random fractions of the data, f=(50,60,70,80,90)%, and calculate the mutual information from these limited samples. If we see the expected simple dependence on the (inverse) number of samples, then we extrapolate to infinite data, and error bars are calculated as the standard deviation from random halves of the data.

We test our estimation procedure in a model with the effective fields $h_{\text{eff}}\left(\left\{\sigma_{\text{j}}\right\}\right)$ are drawn from a normal distribution with zero mean and unit variance, and the distribution over states $P\left(\left\{\sigma_{\text{j}}\right\}\right)$ is Zipf. In Fig. 9(b) we see the expected linear dependence of the information estimate on the inverse number of samples, and the extrapolation matches the exact answer for this model example. We propagate the error to our estimates of the fractional information, $\Delta_{\text{FI}}$. Figure 9(c) shows that the estimate of the FI for each choice of $\sigma_{0}$ comes with a different error. Consequently, we use the median standard deviation, over all choices of $\sigma_{0}$, as the reference to obtain the number of states at the population level (see Fig. 6).

## APPENDIX B: COMPRESSION FOR A POPULATION OF NEURONS IN THE HIPPOCAMPUS

We also have tested compressibility in a population of N=1485 neurons in the mouse hippocampus [27,30]. As in the retinal population, neurons are described with the two states $\sigma_{i}=1(0)$ corresponding to the presence (absence) of activity. In these data the activity is monitored by fluorescent proteins that are sensitive to the intracellular calcium concentration, which provides a slower and coarser readout than direct electrical measurements, but again we can discretize into binary variables.

Neuronal activity in this population is more sparse that in the retinal population, leading to fewer but still a large number of observable states. Describing this population of neurons with a pairwise approximation of the series expansion in Eq. (35) leads to a scatter similar to that observed in the retina with a third order approximation [Fig. 10(a)], although this pairwise approximation is known to capture many collective properties of the system quite accurately [36]. Following the same formalism as before, we calculate the coding cost and the fractional information at the population level, showing that compressibility also is feasible in this population of neurons [Fig. 10(b)]. Our compression approach outperforms the series expansion of $h_{\text{eff}}$ when we compare models with same number of parameters [Fig. 10(c)]. Finally, we implement our iterative coarse-graining algorithm to describe larger populations and find that, as in the retina case, a compression approach exhibits linear growth of number of states that we need (M) as a function of the number of neurons, showing that we can tame the exponential growth [Figs. 10(d) and 10(e)]. We have done our analysis for groups of K=7 and K=8 neurons, finding a similar (and very slow) linear growth [Fig. 10(e)].

## APPENDIX C: DERIVATION OF EQ. (46)

We would like to rewrite the extra visual information carried by a single neuron, Eq. (45), in terms of information shared between that neuron and the network. To do this we follow Ref. [59], and make the various information terms more explicit. We start with the definition:

(C1)
$$\Delta I\left(\sigma_{0};s\right)\equiv I\left(\left\{\sigma_{\text{j}}\right\},\sigma_{0};s\right)-I\left(\left\{\sigma_{\text{j}}\right\};s\right)\;=\left\langle {\text{log}}_{2}\left[\frac{P\left(\left\{\sigma_{\text{j}}\right\},\sigma_{0};s\right)}{P\left(\left\{\sigma_{\text{j}}\right\},\sigma_{0}\right)P(s)}\right]\right\rangle \;-\left\langle {\text{log}}_{2}\left[\frac{P\left(\left\{\sigma_{\text{j}}\right\};s\right)}{P\left(\left\{\sigma_{\text{j}}\right\}\right)P(s)}\right]\right\rangle .$$

We then group the terms and insert factors of unity:

(C2)
$$\Delta I\left(\sigma_{0};s\right)=\left\langle {\text{log}}_{2}\left[\frac{P\left(\left\{\sigma_{\text{j}}\right\},\sigma_{0};s\right)P\left(\left\{\sigma_{\text{j}}\right\}\right)}{P\left(\left\{\sigma_{\text{j}}\right\};s\right)P\left(\left\{\sigma_{\text{j}}\right\},\sigma_{0}\right)}\right]\right\rangle \;=\left\langle {\text{log}}_{2}\left[\frac{P\left(\left\{\sigma_{\text{j}}\right\},\sigma_{0};s\right)P\left(\left\{\sigma_{\text{j}}\right\}\right)}{P\left(\left\{\sigma_{\text{j}}\right\};s\right)P\left(\left\{\sigma_{\text{j}}\right\},\sigma_{0}\right)}\right]\right\rangle$$

(C3)
$$+\left\langle {\text{log}}_{2}\left[\frac{P\left(\sigma_{0}\right)}{P\left(\sigma_{0}\right)}\cdot \frac{P(s)}{P(s)}\cdot \frac{P\left(\sigma_{0}|s\right)}{P\left(\sigma_{0}|s\right)}\right]\right\rangle .$$

Now, we rearrange:

(C4)
$$\Delta I\left(\sigma_{0};s\right)=\left\langle {\text{log}}_{2}\left[\frac{P\left(\sigma_{0}|s\right)P(s)}{P\left(\sigma_{0}\right)P(s)}\right]\right\rangle +\left\langle {\text{log}}_{2}\left[\frac{P\left(\left\{\sigma_{\text{j}}\right\},\sigma_{0}|s\right)}{P\left(\left\{\sigma_{\text{j}}\right\}|s\right)P\left(\sigma_{0}|s\right)}\right]\right\rangle \;+\left\langle {\text{log}}_{2}\left[\frac{P\left(\left\{\sigma_{\text{j}}\right\}\right)P\left(\sigma_{0}\right)}{P\left(\left\{\sigma_{\text{j}}\right\},\sigma_{0}\right)}\right]\right\rangle .$$

Finally, we recognize each of these terms as a mutual information, so that

(C5)
$$\Delta I\left(\sigma_{0};s\right)=I\left(\sigma_{0};s\right)+I\left(\sigma_{0};\left\{\sigma_{\text{j}}\right\}|s\right)-I\left(\sigma_{0};\left\{\sigma_{\text{j}}\right\}\right).$$
